# The *in vitro* and *in vivo* effects of nuclear and cytosolic parafibromin expression on the aggressive phenotypes of colorectal cancer cells: a search of potential gene therapy target

**DOI:** 10.18632/oncotarget.15377

**Published:** 2017-02-16

**Authors:** Hua-chuan Zheng, Jia-jie Liu, Jing Li, Ji-cheng Wu, Lei Yang, Gui-feng Zhao, Xin Zhao, Hua-mao Jiang, Ke-qiang Huang, Zhi-jie Li

**Affiliations:** ^1^ Department of Experimental Oncology and Animal Center, Shengjing Hospital of China Medical University, Shenyang 110004, China; ^2^ Jinzhou Medical University, Jinzhou 121001, China

**Keywords:** colorectal cancer, parafibromin, pathobiological behaviors, aggressive phenotypes, gene therapy

## Abstract

Down-regulated parafibromin is positively linked to the pathogenesis of parathyroid, lung, breast, ovarian, gastric and colorectal cancers. Here, we found that wild-type (WT) parafibromin overexpression suppressed proliferation, tumor growth, induced cell cycle arrest and apoptosis in colorectal cancer cells (p<0.05), but it was the converse for mutant-type (MT, mutation in nucleus localization sequence) parafibromin (p<0.05). Both WT and MT transfectants inhibited migration and invasion, and caused better differentiation (p<0.05) of cancer cells. WT parafibromin transfectants showed the overexpression of Cyclin B1, Cyclin D1, Cyclin E, p38, p53, and AIF in HCT-15 and HCT-116 cells, while MT parafibromin only up-regulated p38 expression. There was lower mRNA expression of *bcl-2* in parafibromin transfectants than the control and mock, while higher expression of *c-myc*, *Cyclin D1*, *mTOR*, and *Raptor*. According to transcriptomic analysis, WT parafibromin suppressed PI_3_K-Akt and FoxO signaling pathways, while MT one promoted PI_3_K-Akt pathway, focal adhesion, and regulation of actin cytoskeleton. Parafibromin was less expressed in colorectal cancer than paired mucosa (p<0.05), and inversely correlated with its differentiation at both mRNA and protein levels (p<0.05). These findings indicated that WT parafibromin might reverse the aggressive phenotypes of colorectal cancer cells and be employed as a target for gene therapy. Down-regulated parafibromin expression might be closely linked to colorectal carcinogenesis and cancer differentiation.

## INTRODUCTION

Parafibromin is a protein encoded by oncosuppressor gene *HRPT2* (hyperparathyroidism 2), whose mutation causes the hyperparathyroidism-jaw tumor syndrome (HPT-JT) and parathyroid cancer. Parafibromin protein can promote the formation of polymerase-associated factor 1 complex, which suppresses RNA polymerase II-mediated general transcription (e.g. *c-myc*, *Cyclin D1* and *β-catenin*), transcriptional elongation, histone H2B ubiquitination, histone H3 methylation, poly (A) length control, coupling of transcriptional and post-transcriptional events [1, 2]. Parafibromin might interact with the ring finger proteins RNF20 and RNF40 to maintain histone 2B monoubiquitination [3]. Parafibromin overexpression was demonstrated to induce G_1_ arrest by repressing cyclin D1 *via* histone H3K9 methylation [4, 5], and cause apoptosis by activating Caspase-3 and Caspase-9, and down-regulating the expression of bcl-2 and survivin [6, 7]. In oral squamous carcinoma cells, oncogenic microRNA-155 down-regulated parafibromin and promoted cell proliferation [8]. Additionally, WT1 overexpression decreased parafibromin level and promoted proliferation by binding to *HRPT2* promoter. Parafibromin overexpression attenuated the protumorigenic activity of WT1 by apoptotic induction [9], promoted IFN-γ-triggered phosphorylation of STAT1 at Tyr (701) by JAKs, and subsequently cellular antiviral response by the interaction with JAK1/2 and STAT1 [10]. In contrast, parafibromin could play an oncogenic role by binding to β-catenin, and thereby activate promitogenic/ Wnt signaling upon tyrosine dephosphorylation by SHP2 [11], which was supported by a positive relationship between parafibromin and ki-67 expression [12].

A novel somatic *HRPT2* missense mutation (Ile60Asn) was identified in the mandibular tumor of an HPT-JT patient. Ile60Asn mutant parafibromin exhibited impaired nucleolar localization, and was less expressed due to an increase in proteasomal degradation. Ile60Asn mutant overexpression led to increased cell proliferation and accumulation in G_2_/M-phase cells [13]. A body of evidences demonstrated that the down-regulated parafibromin expression was positively correlated with the tumorigenesis, aggressive parameters or worse prognoses of parathyroid [14], gastric [15], colorectal [16], breast [17], lung [18], ovarian [7], urothelial [19], head and neck [20] cancers. Previously, we observed parafibromin positivity in the cilia of the fallopian tube [7] and bronchial pseudo-stratified columnar epithelium [18]. In cytosol, parafibromin interacted with muscle α-actinins to promote mobility [21] and with eEF1Bγ and hSki8, which was essential for the destabilization of *p53* mRNA [22]. In the present study, we observed the effects of nuclear and cytosolic parafibromin overexpression on proliferation, apoptosis, senescence, differentiation, glycometabolism, invasion, migration, lamellipodia formation, and tumor growth of colorectal cancer cells, and clarified the relevant mechanisms. In addition, we also analyzed the clinicopathological significances of parafibromin expression in colorectal cancers.

## RESULTS

### The effects of parafibromin overexpression on the phenotypes and relevant molecules of colorectal cancer cells

**Figure 1:**
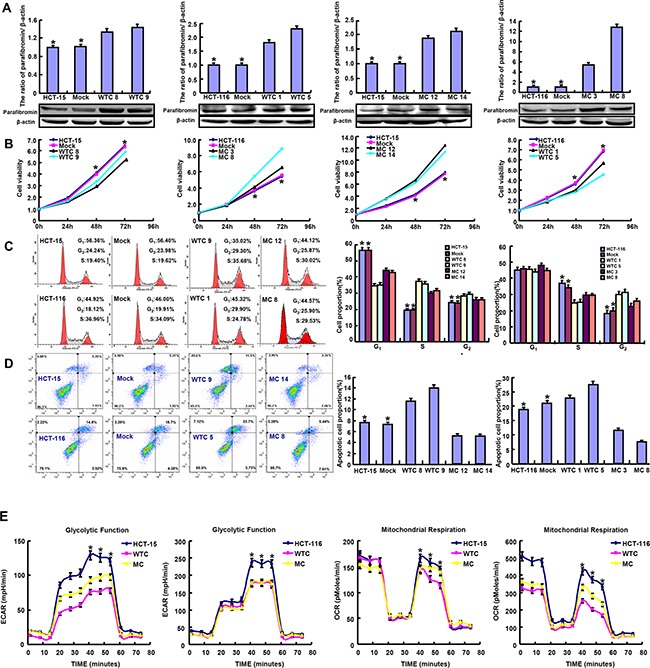
The effects of parafibromin on the proliferation, apoptosis and glycometabolism of colorectal cancer cells Wild-type (WT) and mutant-type (MT) parafibromin expression was confirmed in HCT-15 and HCT-116 transfectants by real-time PCR and Western blot **A**. The cell viability, cell cycle, apoptosis and glucose metabolism of the transfectants were examined by CCK-8 **B**. PI staining **C**. Annexin-V staining **D**. and XF-24 extracellular flux analyzers **E**.

**Figure 2:**
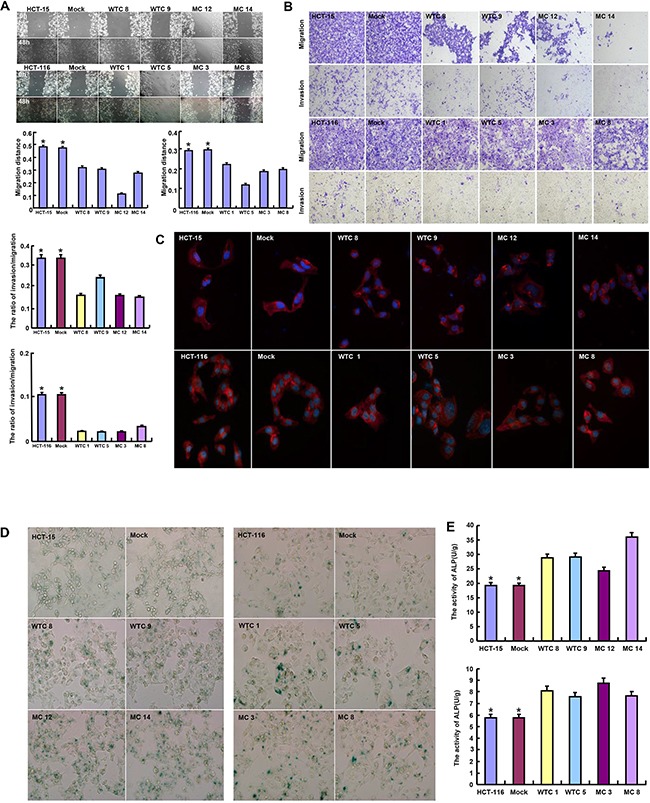
The effects of parafibromin on migration, invasion, senescence and differentiation of colorectal cancer cells The abilities to migrate, invade and form lamellipodia were determined by wound healing assay **A**. transwell chamber assay **B**. and F-actin staining **C**. The senescence and differentiation were detected by β-galactosidase staining **D**. and alkaline phosphotase activity **E**.

**Figure 3:**
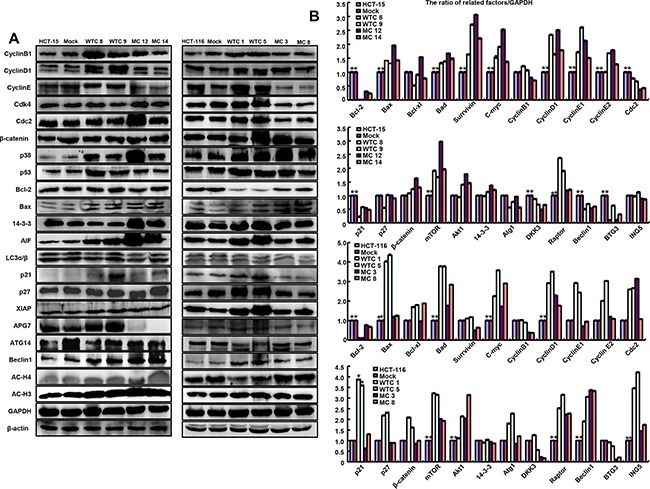
The phenotype-associated molecules were screened in parafibromin transfectants by Western blot **A**. and real-time RT-PCR **B**.

Wild-type (WT) or mutant-type (MT) *HRPT2*-expressing plasmid was successfully transfected into HCT-15 and -116 cells according to the results of real-time RT-PCR and Western blot (Figure 1A). WT parafibromin overexpression resulted in a low proliferation and a high apoptosis in both transfectants, evidenced by CCK-8 and Annexin-V staining (p<0.05), while it was the converse for MT one (Figure 1B and 1C, p<0.05). PI staining showed that WT parafibromin induced S arrest in HCT-15 and G_2_/M arrest in HCT-116, while MT parafibromin had the opposite effects (Figure 1D, p<0.05). There was a weaker glycolysis and mitochondrial respiration in *HRPT2* transfectants than the control and mock (Figure 1E, p<0.05). Both WT and MT *HRPT2* transfectants showed a lower migration and invasion than the control and mock, evidenced by wound healing and transwell assay (Figure 2A and 2B, p<0.05). However, there was no difference in lamellipodia formation and senescence between the control and *HRPT2* transfectants, evidenced by F-actin and β-galactosidase staining (Figure 2C and Figure 3D, p>0.05). WT and MT parafibromin induced the differentiation of HCT-15 and HCT-116 according to alkaline phosphatase (ALP) activity (Figure 2E, p<0.05).

As shown in Figure 3, WT *HRPT2* transfectants showed the overexpression of Cyclin B1, Cyclin D1, Cyclin E, p21, p38, p53, APG7 and AIF in comparison to the control and mock, while MT parafibromin only up-regulated p38 expression. At the mRNA level, there was lower expression of *bcl-2* in *HRPT2* transfectants than the control and mock, while higher expression of *Bad*, *Bax*, *c-myc*, *Cyclin D1*, *mTOR*, and *Raptor*. According to transcriptomic sequencing (Supplementary Figure 1A and 1B; Supplementary Tables 1 and 2), WT parafibromin suppressed PI_3_K-Akt and FoxO signaling pathways. However, MT parafibromin promoted PI_3_K-Akt pathway, focal adhesion, and regulation of actin cytoskeleton (Supplementary Figure 1C-1F; Supplementary Tables 3 and 4).

### The effects of parafibromin overexpression on the tumor growth of colorectal cancer cells in nude mice

**Figure 4:**
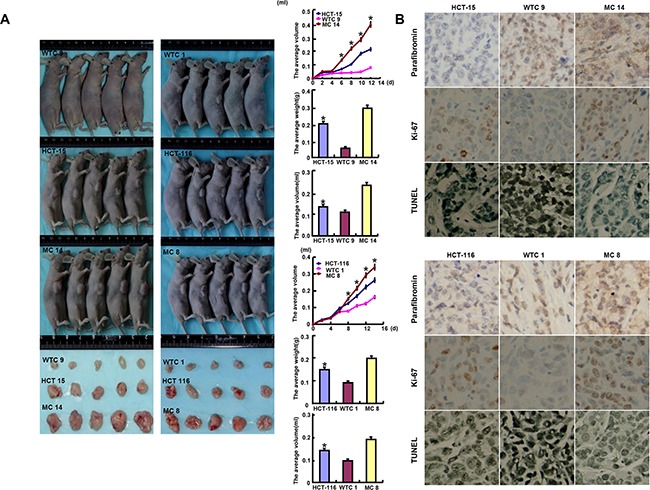
The roles of parafibromin overexpression on the tumor growth of colorectal cancer cells in nude mice The tumor volume and weight were measured by ruling, weighting and capacity measurement **A**. Immunohistochemistry was employed for the detection of parafibromin and ki-67 expression, while TUNEL for apoptotic signal **B**.

HCT-15, HCT-116 and their *HRPT2* transfectants were subcutaneously transplanted into immune-deficient nude mice. As shown in Figure 4, the tumor volume and weight of both parental cells were larger and heavier than those of their WT *HRPT2* transfectants by ruling, weighting and capacity measurement respectively (p<0.05), but smaller and lighter than MT *HRPT2* transfectants (p<0.05). Immunohistochemical data showed WT parafibromin was localized in the nucleus and MT one in the cytoplasm. WT parafibromin suppressed the expression of ki-67 (a marker for proliferation) and induced a strong signal of TUNEL (a marker for apoptosis) in comparison to the control. The converse was observed for MT parafibromin.

### The correlation of parafibromin expression with clinicopathological parameters of colorectal cancers

**Figure 5:**
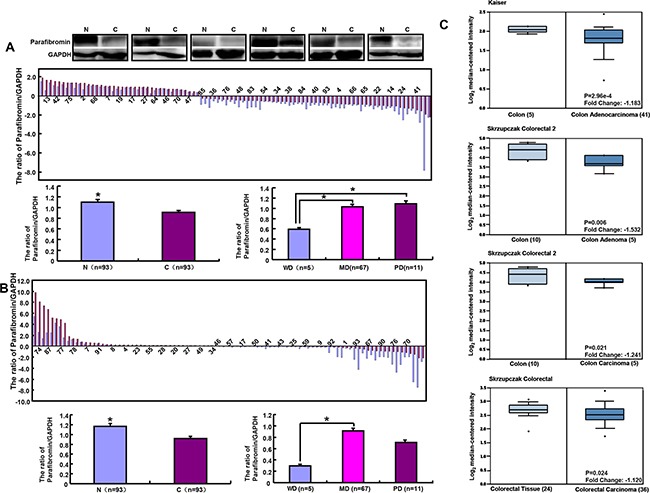
Parafibromin expression in colorectal cancers The mRNA and protein expression of parafibromin was examined and compared with differentiation degree by real-time RT-PCR **A**. and Western blot **B**. respectively. Oncomine's datasets were employed for bioinformatical analysis to compare *HRPT2* mRNA expression with colorectal carcinogenesis **C**. Note: N, normal mucosa; C, cancer; WD, well-differentiated; MD, moderately-differentiated; PD, poorly-differentiated.

Statistically, parafibromin was less expressed in colorectal cancer than paired mucosa according to the optical density (Figure 5A, p<0.05). There was a weaker expression of parafibromin in well- differentiated than moderately- or poorly-differentiated adenocarcinomas (Figure 5A, p<0.05). At the mRNA level, *HRPT2* expression was more detectable in colorectal mucosa than adjacent cancer (Figure 5B, p<0.05). Well-differentiated adenocarcinoma less expressed *HRPT2* than moderately-differentiated ones (Figure 5B, p<0.05). Then, we used Kaiser's and Skrzypczak's datasets to perform bioinformatical analysis and found that *HRPT2* mRNA expression was higher in colorectal normal mucosa than adenoma or cancer (Figure 5C, p<0.05).

## DISCUSSION

In agreement with the immunohistochemical findings from gastric and colorectal cancers, and RT-PCR data in lung cancer [15, 16, 18], we found that a lower expression of *HRPT2* mRNA and its encoding protein in colorectal cancer than matched mucosa according to RT-PCR, Western blot and bioinformatical analysis, supporting the opinion that parafibromin functions as a tumor suppressor. Our *in vitro* experiment showed that both WT and MT parafibromin overexpression caused the differentiation of colorectal cancer cells, evidenced by enhanced ALP activity. However, the higher expression of parafibromin mRNA and protein was for first time observed in moderately- than well-differentiated adenocarcinomas, inconsistent with the data in gastric cancer [15]. The discrepancy might be due to the different approaches and tissue specificity.

Immunohistochemically, parafibromin protein was observed in the cilia of bronchial pseudostratified columnar [18] or fallopian tube epithelium [7]. Reportedly, cytosolic parafibromin interacted with muscle actinin-2 and actinin-3 [21]. To verify the effects of cytosolic parafibromin on aggressiveness, we transfected MT *HRPT2* (mutation in nuclear localization signal) into colorectal cancer cells, and found that it might enhance the proliferation and suppress the apoptosis. IP staining demonstrated that MT parafibromin overexpresison caused S or G_2_ progression, opposite to the data of WT one [4]. In contrast, MT parafibromin also reduced the migrative and invasive capacities of colorectal cancer cells with no difference in lamellipodia formation. Therefore, we speculated that cytosolic parafibromin protein couldn't interact with actinins in colorectal cancer cells. *In vivo* tumor-bearing model showed that MT parafibromin promoted the growth by enhancing proliferation and suppressing apoptosis. On the other hand, WT had the opposite results to MT ones about proliferation, apoptosis and tumor growth, while it was the same as migration and invasion. Therefore, WT parafibromin could be employed as a molecular target of gene therapy to suppress the tumor growth and metastasis in the treatment of colorectal cancers.

Cyclin-Cdk complex promotes cell cycle progression through G_1_ and Cyclin B1 is helpful for the mitosis by interacting with Cdk1 [23]. Therefore, Cyclin D1, E and B1 overexpression was positively linked to WT parafibromin-mediated G_2_ arrest of colorectal cancer cells. However, Cyclin E hypoexpression indicated that MT parafibromin-mediated G_2_ progression was independent of Cyclin E in colorectal cancer cells. No change in β-catenin expression suggested that both WT and MT parafibromin might not target Wnt/β-catenin signal pathway. Reportedly, activated p38 MAP kinase by phosphorylation at Thr-180 and Tyr-182 has been shown to phosphorylate and activate MAPKAP kinase 2 and to phosphorylate the transcription factors (ATF2, Mac and MEF2) [24]. Higher phospho-p38 level in colorectal cancer cell transfectants indicated that cytoplasmic and nuclear parafibromin might regulate the phosphorylation of p38 possibly by distinct pathways. p53 is a master switch that coordinates stress signals associated with apoptosis and cell cycle arrest, but it is translationallly regulated by WT parafibromin [22]. AIF might initiate a Caspase-independent pathway of apoptosis by causing DNA fragmentation and chromatin condensation and increasing the permeability of the mitochondrial membrane upon apoptosis [25]. WT parafibromin up-regulated AIF and p53 expression in colorectal cancer cells, which induced apoptosis. Strangely, AIF overexpression was also detectable in MT parafibromin transfectants of HCT-15 with apoptotic resistance, which should be investigated in the future.

Reportedly, parafibromin located in the nucleus can induce apoptosis and G_1_ phase arrest in osteosarcoma cells and suppressed the MEK/ERK and PI_3_K/AKT signaling pathways [6]. Here, we performed transcrpitomic sequencing and bioinformatical analysis to screen the signal pathways of WT and MT parafibromin in colorectal cancer cells. WT parafibromin overexpression was found to suppress PI_3_K-Akt and FoxO signaling pathways in both HCT-15 and -116 cells, which might be closely linked to its anti-cancer effects. In contrast, MT parafibromin overexpression promoted PI_3_K-Akt signaling pathway, focal adhesion, and regulation of actin cytoskeleton, opposite to the inhibitory effects of MT parafibromin in colorectal cancer cells. The discrepancy might be due to other signal pathways involved in the biological event and the far distance from transcriptional, translational to biological effects, which will be further investigated. Additionally, the different subcellular localization and co-factors of proteins also influenced the migration and invasion of colorectal cancer cells.

In summary, WT parafibromin might reverse the aggressive phenotypes of colorectal cancer cells and be employed as a target for the gene therapy. Its cytosolic localization might partially worsen the aggressiveness. Down-regulated parafibromin expression might be closely linked to colorectal carcinogenesis and cancer differentiation.

## MATERIALS AND METHODS

### Cell culture and transfection

HCT-15 and HCT-116 were maintained in RPMI 1640 medium supplemented with 10% fetal bovine serum (FBS), 100 units/ml penicillin and 100 μg/ml streptomycin in a humidified atmosphere of 5% CO_2_ at 37°C. The cells were transfected with psmpvw-wt- parafibromin (wild-type, WT) or psmpvw-126a/139a-parafibromin (mutant-type, MT) plasmid at 70% confluence after seeding on dishes using lipid method according to the manufacturer's instructions (QIAGEN, USA). All cells were harvested by centrifugation, rinsed with PBS, and subjected to total protein or RNA extraction.

### Proliferation assay

Cell Counting Kit-8 (CCK-8, Dojindo, Japan) was employed to determine the number of viable cells. Cell viability curve was made to confirm cell proliferation.

### Cell cycle analysis

Cells were harvested, washed by PBS and fixed in cold ethanol for 4 h at -20°C. After washed by PBS, the cells were immersed with 1mL RNase (0.25 mg/mL) at 37°C for 1 h. The cells were pelleted, resuspended in propidium iodide (PI, 50μg/mL), and incubated in the dark for 30 min. Finally, flow cytometry was carried out to detect PI signal.

### Apoptosis assay by flow cytometry

Flow cytometry was performed with PI and FITC-labeled annexin V (BD Pharmingen, USA) to detect phosphatidylserine externalization (on the surface of cell membrane) as an endpoint indicator of apoptosis as the recommendation.

### Wound healing assay

Cells were seeded at a density of 1.0×10^6^ cells/well in 6-well culture plates. After they had grown to confluence, the cell monolayer was scraped with a pipette tip to create a scratch, washed by PBS for three times and cultured in the FBS-free medium. Cells were photographed at 48 h and the scratch area was measured using Image J software.

### Transwell chamber assay

For invasion assay, 2.5 × 10^5^ cells were resuspended in serum-free RPMI 1640, and seeded in the matrigel-coated insert on the top portion of the chamber. The lower compartment of the chamber contained 10% v/v FBS as a chemoattractant. After incubated at 37°C, 5% CO_2_ for 24 h, cells on the membrane were scrubbed, washed with PBS and fixed in 100% methanol and stained with Giemsa dye for the measurement. For migration assay, the procedures were the same as described above excluding the control-membrane insert.

### Immunofluorescence

Cells were grown on glass coverslips, washed twice with PBS, fixed with 4% formaldehyde for 10 min, and permeabilized with 0.25% Triton X-100 for 10 min. After washing with PBS, cells were incubated with Alexa Fluor® 568 phalloidin (invitrogen) for 1 h. After that, the sections were mounted with VECTASHIELD mounting medium with DAPI (Vector Laboratories). Finally, the microphotography was performed under the fluorescence microscopy.

### β-galactosidase staining

β-galactosidase staining was performed with a senescence-+associated β-galactosidase staining kit (Beyotime, China). Finally, cells were observed under a light inverted microscope.

### Alkaline phosphatase (ALP)

ALP activity was used as an additional marker of the degree of colorectal differentiation. The cells were harvested, washed, broken and subjected to the determination of ALP activity using the Sigma Diagnostics ALP reagent (Sigma, USA). The protein content of the samples was determined using Coomassie Protein Assay Reagent Kit (Biorad, USA). ALP activity was calculated as activity units per g of protein.

### Metabolism assays

Oxygen consumption rates and extracellular acidification rates were measured in XF media (nonbuffered RPMI 1640 containing either 10mM or 25 mM glucose or galactose, 2 mM L-glutamine, and 1 mM sodium pyruvate) under basal conditions and in response to mitochondrial inhibitors, 1 mM oligomycin and/or 100 nM rotenone + 1 mM antimycin A (Sigma) on the XF-24 Extracellular Flux Analyzers (Seahorse Bioscience). ATP measurements were performed with ATP determination kit (Invitrogen) and glucose concentrations were measured with a glucose assay kit (Eton Bioscience Inc.).

### Subjects

Colorectal cancers and matched non-neoplastic mucosa (n=93) were collected from the surgical resection in our hospital (Shenyang) and frozen in -80°C until protein and RNA extraction between Jan 2014 and Dec 2015. The patients with CRC were 49 men and 44 women (42~84years, mean=63.4 years). Among them, 47 cases are accompanied with lymph node metastasis. Histologically, there were 5 well-, 67 moderately- and 11 poorly-differentiated adenocarcinomas. None of the patients underwent chemotherapy, radiotherapy or adjuvant before surgery. They all provided consent for use of tumor tissue for clinical research and the ethical committee of our hospital approved the research protocol.

### Xenograft models

Female Balb/c nude (nu/nu) mice of 6-8 weeks were bred and used for implantation. The animals were maintained under specific pathogen-free conditions, and food and water were supplied ad libitum. Housing and all procedures involving animals were performed according to protocols in compliance with the committee for animal experiments guidelines on animal welfare of China Medical University. Subcutaneous xenografts were established by injection of 1× 10^6^ cancer cells/mouse to the axilla (n=10/group). Tumor growth was then monitored for 12 days. For each tumor, measurements were made using calipers, and tumor volumes were calculated as follows: width ^2^ ×depth× 0.52. Until the end of the experiment, the mice were randomly selected to be anesthetized, sacrificed, photographed, and subjected to weighting and capacity measurement. The part of tumors were subsequently fixed in 4% paraformaldehyde for 24 h, and then embedded in paraffin.

### Preparation of RNA-seq libraries, sequencing and data analysis

The total RNA samples were firstly treated with DNase I to degrade any possible DNA contamination. Then, mRNA was enriched by the oligo (dT) magnetic beads. Mixed with the fragmentation buffer, mRNA was fragmented into short fragments and converted into the first strand of cDNA using random hexamer-primer. Buffer, dNTPs, RNase H and DNA polymerase I were added to synthesize the double strand cDNA, which was purified with magnetic beads. End preparation and 3′-end single adenine addition was then performed. Finally, sequencing adaptors were ligated to the fragments, followed by PCR amplification. The library products are subjected to sequencing via Illumina HiSeq^TM^ 2000.

Raw data was subjected to quality control and reads filtration, and aligned to the reference sequences. The alignment data was utilized to calculate distribution of reads on reference genes and mapping ratio. Subsequently, we proceed with downstream analysis including gene expression and deep analysis based on gene expression (PCA/correlation /screening differentially expressed genes and so on). Further, we performed deep analysis based on DEGs, including Gene Ontology enrichment analysis, and KEGG pathway enrichment analysis.

### Real-time RT-PCR

Total RNA was extracted from colorectal cancer cells and tissues using RNeasy mini kit (QIAGEN, Germany). Two micrograms of total RNA was subjected to cDNA synthesis using avian myeloblastosis virus transcriptase and random primer (Takara, Japan). The primers were listed in Supplementary Table 5. Real-time PCR was carried out according to the protocol of SYBR Premix Ex Taq^TM^ II kit (Takara) in 20μL mixture. The expression level was expressed as 2^−ΔCt^, where ΔCt =Ct (gene)–Ct (GAPDH). Additionally, the expression level of the control was considered as “1”.

### Western blot

Protein assay were performed using Biorad protein assay kit. The denatured protein was separated on 10% SDS-polyacrylamide gel and transferred to Hybond membrane (Amersham, Germany), which was then blocked overnight in 5% milk in TBST. For immunobloting, the membrane was incubated for 1 h with primary antibodies (Supplementary Table 6). Then, it was rinsed by TBST and incubated with anti-mouse, anti-rabbit or anti-goat IgG conjugated to horseradish peroxidase (DAKO, USA) for 1 h. Bands were visualized by ECL-Plus detection reagents (Santa cruz, USA). The densitometry quantification was performed with a β-actin or GAPDH as a control using Scion Image.

### Immunohistochemistry

The immunohistochemistry was performed according to the procedures recommended by Kumada et al. [26]. The mouse anti-parafibromin or rabbit anti-ki-67 antibody were purchased from Santa cruz and DAKO respectively. Immunoreactivity for parafibromin and ki-67 was localized in the nucleus.

### Terminal digoxigenin-labeled dUTP nick-end labeling (TUNEL)

Cell apoptosis was assessed using TUENL, a method that is based on the specific binding O-TdT to the 3-OH ends of DNA, ensuring the synthesis of a polydeoxynucleotide polymer. For this purpose, ApopTag Plus Peroxidase In Situ Apoptosis Detection Kit (Millipore) was employed according to the recommendation. Omission of the working strength TdT enzyme was considered as negative control.

### Oncomine analysis

The individual gene expression level of *HRPT2* was analyzed using Oncomine (www.oncomine.org), a cancer microarray database and web-based data mining platform for a new discovery from genome-wide expression analyses. We compared the differences in *HRPT2* mRNA level between colorectal mucosa and adenoma/cancer. All data were log-transformed, median centered per array, and standard deviation normalized to one per array.

### Statistical analysis

Statistical evaluation was performed using student t test to compare the means of different groups. p<0.05 was considered as statistically significant. SPSS 10.0 software was employed to analyze all data.

## SUPPLEMENTARY FIGURE AND TABLES




