# Supplementary material for: The in vitro and in vivo effects of nuclear and cytosolic parafibromin expression on the aggressive phenotypes of colorectal cancer cells: a search of potential gene therapy target
Source: Oncotarget. 2017 Feb 16;8(14):23603–12. doi: 10.18632/oncotarget.15377 (PMC5410330; doi:10.18632/oncotarget.15377)
Supplement: Supplementary file 1 [file oncotarget-08-23603-s001.pdf]

The *in vitro* and *in vivo* effects of nuclear and cytosolic parafibromin expression on the aggressive phenotypes of colorectal cancer cells: a search of potential gene therapy target

SUPPLEMENTARY FIGURE AND TABLES

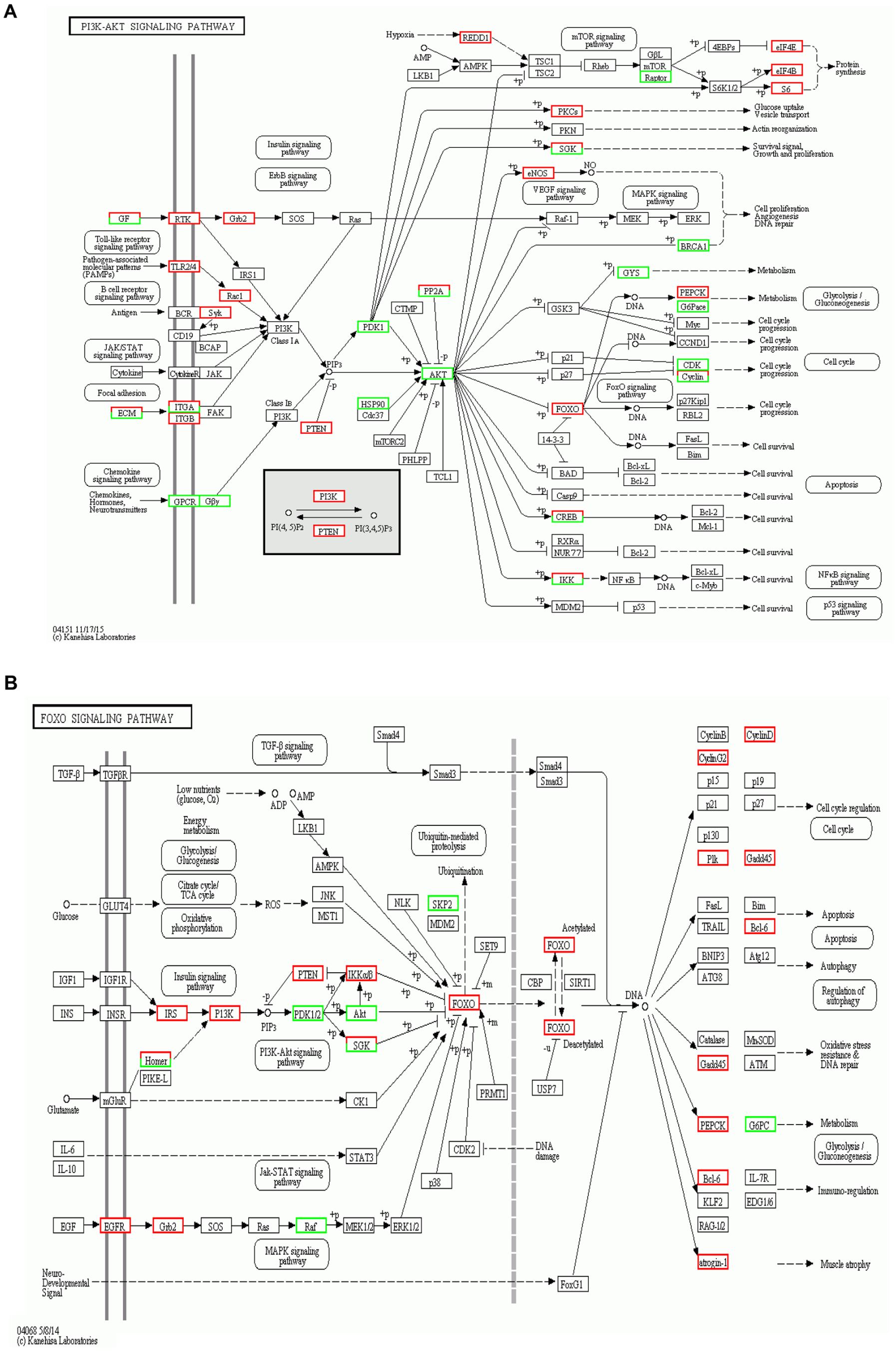

Supplementary Figure 1: Pathways of altered genes regulated by wild- and mutant-type parafibromin expression in colorectal cancer cells. The significantly altered pathways were observed in HCT-15 overexpressing wild-type parafibromin **A.** and **B.** and in HCT-116 overexpressing mutant-type parafibromin. (*Continued*)

C

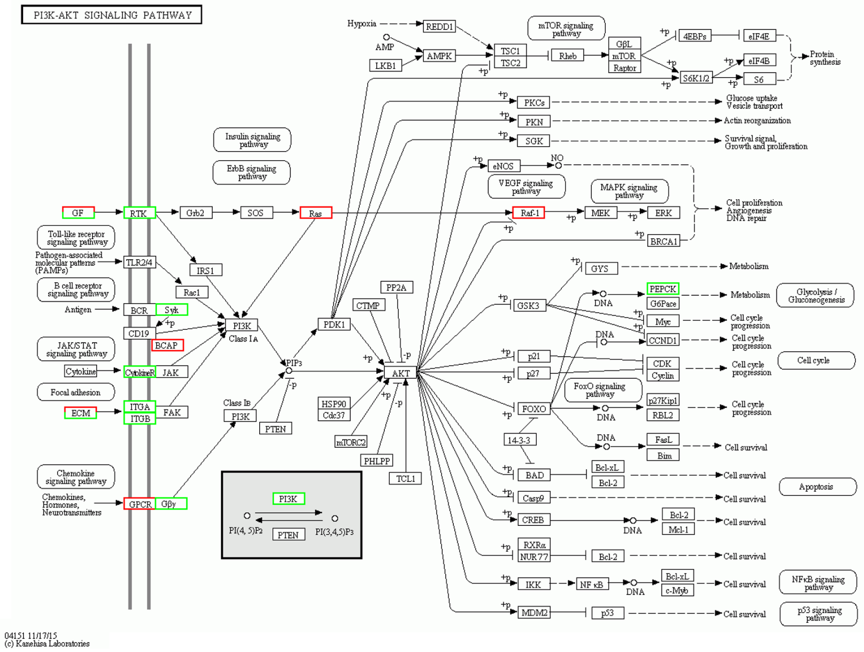

D

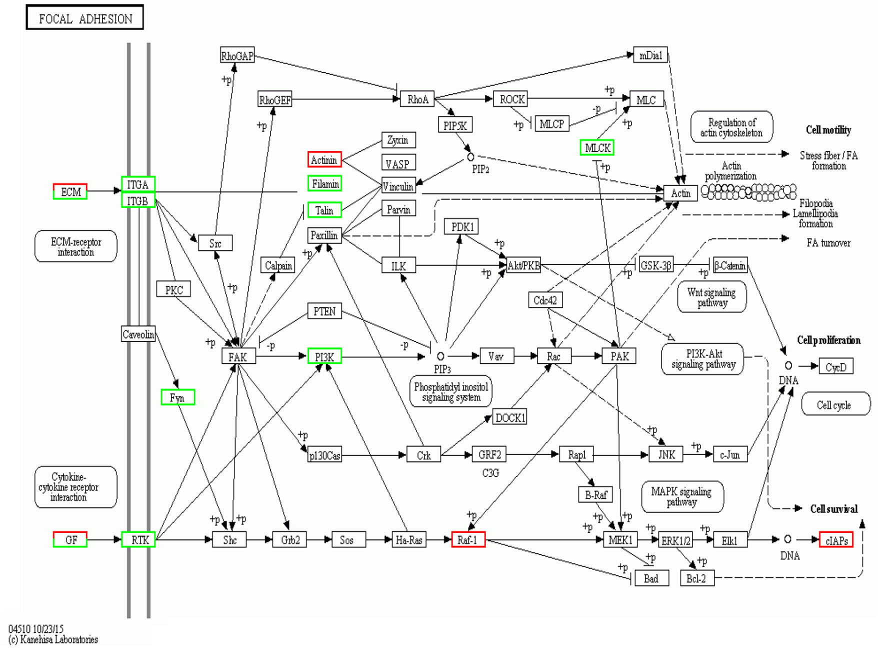

Supplementary Figure 1: (*Continued*) Pathways of altered genes regulated by wild- and mutant-type parafibromin expression in colorectal cancer cells. C, and **D.** The red fame represents up-regulated genes and the green frame represents down-regulated ones. (*Continued*)

E

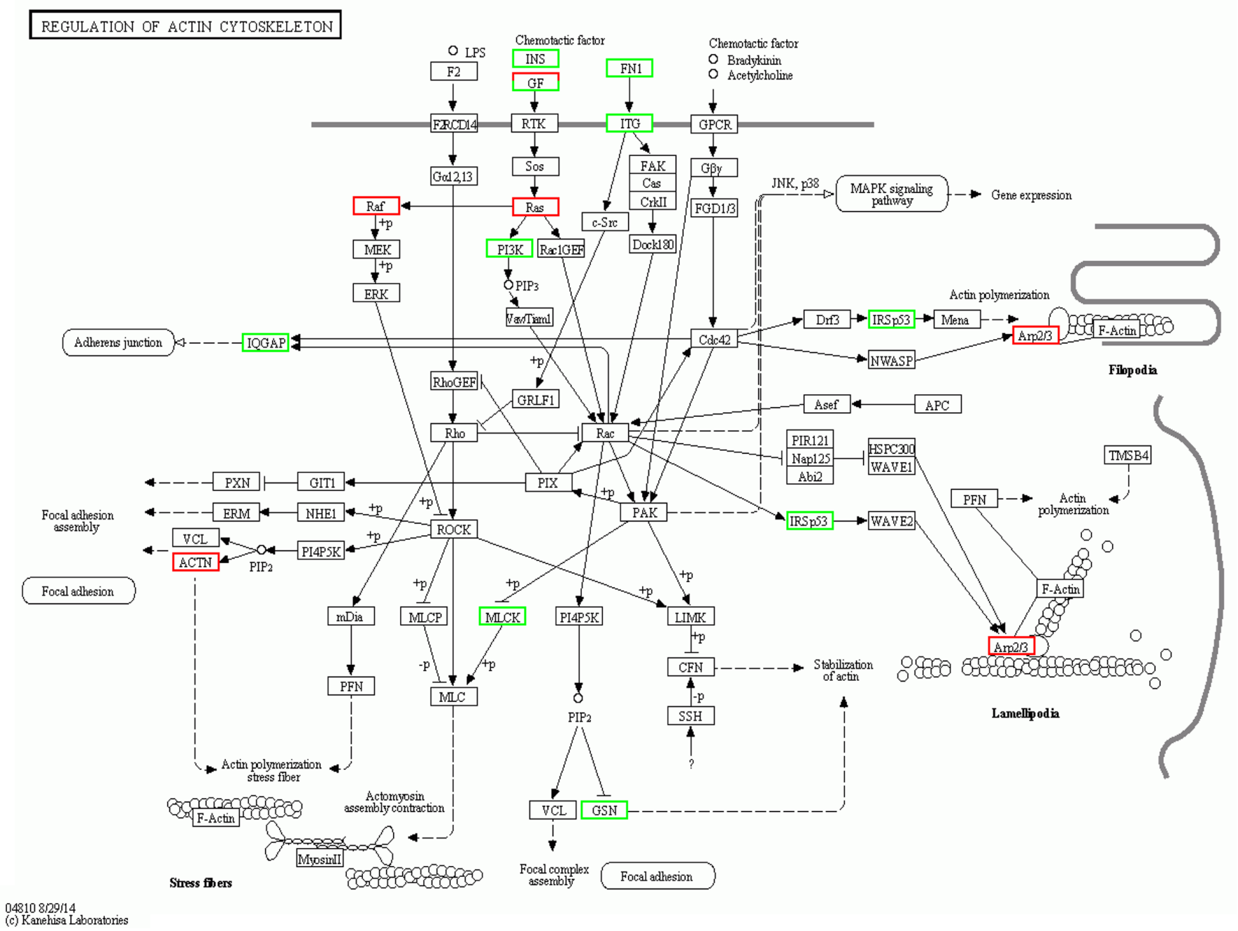

Supplementary Figure 1: (*Continued*) Pathways of altered genes regulated by wild- and mutant-type parafibromin expression in colorectal cancer cells. E. The red fame represents up-regulated genes and the green frame represents down-regulated ones.

Supplementary Table 1: The significantly altered signal pathways was listed in HCT-15 overexpressing wild-type parafibromin

Num	Pathway	DEGs	All genes	p value
1	Transcriptional misregulation in cancer	188 (10.4%)	1471 (8.53%)	0.00187546
2	Arginine and proline metabolism	38 (2.1%)	228 (1.32%)	0.002648931
3	MicroRNAs in cancer	47 (2.6%)	297 (1.72%)	0.002667464
4	Fanconi anemia pathway	16 (0.88%)	75 (0.43%)	0.004235746
5	Herpes simplex infection	43 (2.38%)	285 (1.65%)	0.009181581
6	MAPK signaling pathway	57 (3.15%)	399 (2.31%)	0.009613304
7	Glycosylphosphatidylinositol-anchor biosynthesis	9 (0.5%)	36 (0.21%)	0.01025868
8	Proteoglycans in cancer	63 (3.48%)	450 (2.61%)	0.01037147
9	Metabolic pathways	338 (18.69%)	2889 (16.75%)	0.01105844
10	Purine metabolism	168 (9.29%)	1368 (7.93%)	0.01451053
11	Inositol phosphate metabolism	18 (1%)	100 (0.58%)	0.01539639
12	Ribosome biogenesis in eukaryotes	21 (1.16%)	123 (0.71%)	0.01672182
13	Phagosome	49 (2.71%)	351 (2.03%)	0.02274323
14	Ras signaling pathway	48 (2.65%)	343 (1.99%)	0.02304767
15	Osteoclast differentiation	32 (1.77%)	213 (1.23%)	0.02344294
16	Homologous recombination	9 (0.5%)	41 (0.24%)	0.02376233
17	mTOR signaling pathway	19 (1.05%)	113 (0.65%)	0.02553419
18	Adipocytokine signaling pathway	18 (1%)	107 (0.62%)	0.02903749
19	PI3K-Akt signaling pathway	72 (3.98%)	553 (3.21%)	0.03072005
20	FoxO signaling pathway	27 (1.49%)	178 (1.03%)	0.03153294
21	Phenylalanine metabolism	7 (0.39%)	32 (0.19%)	0.04445025
22	Non-small cell lung cancer	16 (0.88%)	97 (0.56%)	0.04447518
23	Hepatitis B	32 (1.77%)	225 (1.3%)	0.04575855
24	ErbB signaling pathway	22 (1.22%)	145 (0.84%)	0.04839595

Supplementary Table 2: The significantly altered signal pathways was listed in HCT-15 overexpressing mutant-type parafibromin

Num	Pathway	DEGs	All genes	p value
1	Signaling pathways regulating pluripotency of stem cells	13 (3.68%)	204 (1.18%)	0.0003045876
2	NF-kappa B signaling pathway	10 (2.83%)	153 (0.89%)	0.00122431
3	Wnt signaling pathway	12 (3.4%)	216 (1.25%)	0.001696107
4	Focal adhesion	20 (5.67%)	477 (2.76%)	0.002039501
5	Pathways in cancer	24 (6.8%)	644 (3.73%)	0.003569215
6	Drug metabolism - cytochrome P450	6 (1.7%)	73 (0.42%)	0.003785045
7	Chemical carcinogenesis	7 (1.98%)	101 (0.59%)	0.004704071
8	PI3K-Akt signaling pathway	21 (5.95%)	553 (3.21%)	0.005057751
9	Amoebiasis	11 (3.12%)	222 (1.29%)	0.00616262
10	Notch signaling pathway	6 (1.7%)	81 (0.47%)	0.006301151
11	Fatty acid biosynthesis	3 (0.85%)	19 (0.11%)	0.006452852
12	p53 signaling pathway	8 (2.27%)	135 (0.78%)	0.006640105
13	Metabolism of xenobiotics by cytochrome P450	6 (1.7%)	85 (0.49%)	0.007936326
14	MAPK signaling pathway	16 (4.53%)	399 (2.31%)	0.00833879
15	Regulation of actin cytoskeleton	21 (5.95%)	588 (3.41%)	0.009851956
16	HTLV-I infection	15 (4.25%)	376 (2.18%)	0.01092573
17	Complement and coagulation cascades	9 (2.55%)	181 (1.05%)	0.01237247
18	Phospholipase D signaling pathway	11 (3.12%)	247 (1.43%)	0.01309616
19	Epithelial cell signaling in Helicobacter pylori infection	6 (1.7%)	95 (0.55%)	0.01332536
20	Basal cell carcinoma	5 (1.42%)	69 (0.4%)	0.01346179
21	Salmonella infection	13 (3.68%)	317 (1.84%)	0.01394383
22	MicroRNAs in cancer	12 (3.4%)	297 (1.72%)	0.01969981
23	Cyanoamino acid metabolism	2 (0.57%)	11 (0.06%)	0.02032378
24	Rap1 signaling pathway	14 (3.97%)	382 (2.21%)	0.02618435
25	FoxO signaling pathway	8 (2.27%)	178 (1.03%)	0.03034572
26	Rheumatoid arthritis	6 (1.7%)	115 (0.67%)	0.03087116
27	Cocaine addiction	5 (1.42%)	91 (0.53%)	0.03891136
28	Proteoglycans in cancer	15 (4.25%)	450 (2.61%)	0.04456468
29	Taurine and hypotaurine metabolism	2 (0.57%)	17 (0.1%)	0.0463759
30	Pertussis	6 (1.7%)	127 (0.74%)	0.04647301
31	Circadian rhythm	3 (0.85%)	40 (0.23%)	0.04809626
32	Dorso-ventral axis formation	4 (1.13%)	67 (0.39%)	0.04831849

Supplementary Table 3: The significantly altered signal pathways was listed in HCT-116 overexpressing wild-type parafibromin

Num	Pathway	DEGs	All genes	p value
1	Steroid biosynthesis	5 (2.75%)	31 (0.18%)	1.682023e-05
2	Terpenoid backbone biosynthesis	4 (2.2%)	29 (0.17%)	0.0002315632
3	TNF signaling pathway	8 (4.4%)	166 (0.96%)	0.0003846124
4	AMPK signaling pathway	8 (4.4%)	202 (1.17%)	0.001385177
5	Hepatitis B	8 (4.4%)	225 (1.3%)	0.002716428
6	Prostate cancer	6 (3.3%)	151 (0.88%)	0.005360733
7	NF-kappa B signaling pathway	6 (3.3%)	153 (0.89%)	0.005710005
8	Insulin resistance	6 (3.3%)	157 (0.91%)	0.00645804
9	HTLV-I infection	10 (5.49%)	376 (2.18%)	0.006702799
10	Hippo signaling pathway	7 (3.85%)	216 (1.25%)	0.008095849
11	Aldosterone synthesis and secretion	5 (2.75%)	135 (0.78%)	0.01426429
12	PI3K-Akt signaling pathway	12 (6.59%)	553 (3.21%)	0.0145038
13	Prolactin signaling pathway	4 (2.2%)	94 (0.54%)	0.01751813
14	Viral carcinogenesis	8 (4.4%)	334 (1.94%)	0.02562385
15	Toxoplasmosis	5 (2.75%)	161 (0.93%)	0.02807218
16	Amoebiasis	6 (3.3%)	222 (1.29%)	0.03054104
17	Rheumatoid arthritis	4 (2.2%)	115 (0.67%)	0.03356443
18	Sphingolipid metabolism	3 (1.65%)	68 (0.39%)	0.03511706
19	Maturity onset diabetes of the young	2 (1.1%)	29 (0.17%)	0.03729636
20	Fatty acid metabolism	3 (1.65%)	71 (0.41%)	0.03915255
21	FoxO signaling pathway	5 (2.75%)	178 (1.03%)	0.0405473
22	Biosynthesis of unsaturated fatty acids	2 (1.1%)	32 (0.19%)	0.04464206
23	Vasopressin-regulated water reabsorption	3 (1.65%)	75 (0.43%)	0.04488299
24	Amphetamine addiction	4 (2.2%)	128 (0.74%)	0.04670295
25	Chagas disease (American trypanosomiasis)	4 (2.2%)	130 (0.75%)	0.04894468

Supplementary Table 4: The significantly altered signal pathways was listed in HCT-116 overexpressing mutant-type parafibromin

Num	Pathway	DEGs	All genes	p value
1	Terpenoid backbone biosynthesis	6 (1.28%)	29 (0.17%)	0.0001090895
2	Tuberculosis	20 (4.26%)	331 (1.92%)	0.0007692985
3	PI3K-Akt signaling pathway	28 (5.97%)	553 (3.21%)	0.00125251
4	B cell receptor signaling pathway	11 (2.35%)	139 (0.81%)	0.001466404
5	Pathways in cancer	29 (6.18%)	644 (3.73%)	0.005622915
6	Melanoma	8 (1.71%)	101 (0.59%)	0.006263179
7	Hippo signaling pathway	13 (2.77%)	216 (1.25%)	0.006324225
8	Type I diabetes mellitus	6 (1.28%)	63 (0.37%)	0.007181131
9	HTLV-I infection	19 (4.05%)	376 (2.18%)	0.007376367
10	Amoebiasis	13 (2.77%)	222 (1.29%)	0.007889762
11	Steroid biosynthesis	4 (0.85%)	31 (0.18%)	0.009486949
12	Dorso-ventral axis formation	6 (1.28%)	67 (0.39%)	0.009636588
13	Complement and coagulation cascades	11 (2.35%)	181 (1.05%)	0.01075555
14	Melanogenesis	9 (1.92%)	134 (0.78%)	0.01101561
15	Focal adhesion	22 (4.69%)	477 (2.76%)	0.01146811
16	Prolactin signaling pathway	7 (1.49%)	94 (0.54%)	0.01412764
17	ECM-receptor interaction	14 (2.99%)	269 (1.56%)	0.01570733
18	Cholinergic synapse	9 (1.92%)	144 (0.83%)	0.01698194
19	Phospholipase D signaling pathway	13 (2.77%)	247 (1.43%)	0.01793842
20	Regulation of actin cytoskeleton	25 (5.33%)	588 (3.41%)	0.01870932
21	Endocytosis	20 (4.26%)	449 (2.6%)	0.02161125
22	Prostate cancer	9 (1.92%)	151 (0.88%)	0.02237353
23	Notch signaling pathway	6 (1.28%)	81 (0.47%)	0.0228868
24	Ovarian steroidogenesis	5 (1.07%)	60 (0.35%)	0.02334305
25	Signaling pathways regulating pluripotency of stem cells	11 (2.35%)	204 (1.18%)	0.02404366
26	Ras signaling pathway	16 (3.41%)	343 (1.99%)	0.02591466
27	GABAergic synapse	8 (1.71%)	131 (0.76%)	0.02674136
28	Colorectal cancer	6 (1.28%)	84 (0.49%)	0.02680841
29	Tight junction	19 (4.05%)	436 (2.53%)	0.03002971
30	Serotonergic synapse	9 (1.92%)	160 (0.93%)	0.030978
31	MicroRNAs in cancer	14 (2.99%)	297 (1.72%)	0.03306778
32	D-Arginine and D-ornithine metabolism	2 (0.43%)	11 (0.06%)	0.03448199
33	Cyanoamino acid metabolism	2 (0.43%)	11 (0.06%)	0.03448199
34	Glutathione metabolism	11 (2.35%)	221 (1.28%)	0.03964747
35	Arginine and proline metabolism	11 (2.35%)	228 (1.32%)	0.04775865

Supplementary Table 5: Primers employed in the present study.

See Supplementary File 1

Supplementary Table 6: Primary antibodies employed in the present study

Names	Source	Company
Cyclin B1 (GNS1)	Mouse	Santa Cruz Biotech. Inc.
Cyclin D1 (H-295)	Rabbit	Santa Cruz Biotech. Inc.
CyclinE (HE12)	Rabbit	Santa Cruz Biotech. Inc.
Cdc2 (B-6)	Mouse	Santa Cruz Biotech. Inc.
Cdk4 (C-22)	Rabbit	Santa Cruz Biotech. Inc.
p53 (FL-393)	Rabbit	Santa Cruz Biotech. Inc.
Bcl-2 (C 21)	Rabbit	Santa Cruz Biotech. Inc.
Bax (B-9)	Mouse	Santa Cruz Biotech. Inc.
AIF(E-1)	Mouse	Santa Cruz Biotech. Inc.
XIAP(H-202)	Rabbit	Santa Cruz Biotech. Inc.
Beclin 1	Rabbit	Abcam
Apg7(N-20)	Goat	Santa Cruz Biotech. Inc.
β-catenin(C-18)	Goat	Santa Cruz Biotech. Inc.
p21 (F-5)	Mouse	Santa Cruz Biotechnology
p27	Mouse	Santa Cruz Biotechnology
P38α/β(H-147)	Rabbit	Santa Cruz Biotech. Inc.
Ac-histone 3 (Lys 9/14)	Goat	Santa Cruz Biotechnology
Ac-histone 4 (Lys 8)	Rabbit	Santa Cruz Biotechnology
14-3-3 (H-8)	Mouse	Santa Cruz Biotechnology
Atg14	Rabbit	cell signaling
LC3α/β	Rabbit	Wanleibio
β-actin(C-4)	Mouse	Santa Cruz Biotech. Inc.
GAPDH	Rabbit	Wanleibio
