# Supplementary material for: The in vitro and in vivo effects of nuclear and cytosolic parafibromin expression on the aggressive phenotypes of colorectal cancer cells: a search of potential gene therapy target
Source: Oncotarget. 2017 Feb 16;8(14):23603–12. doi: 10.18632/oncotarget.15377 (PMC5410330; doi:10.18632/oncotarget.15377)
Supplement: Supplementary file 2 [file oncotarget-08-23603-s002.docx]

**Supplementary Table 5. Primers employed in the present study**

| **Names** | **Primer‘ s sequence** | **Distribution** | **AT(^o^C)** | **Product size(bp)** | **Extension time(s)** |
| --- | --- | --- | --- | --- | --- |
| *Cyclin B1* | F: 5' -GTTATGCAGCACCTG-3' | NM_001088590 | 60 | 150 | 34 |
|  | R: 5'-CTTGGCTAAATCTTGAACT-3' | 1388-1537 |  |  |  |
| *CyclinE1* | F: 5'-GGATGTTGACTGCCTTGA-3' | BA000005 | 60 | 107 | 34 |
|  | R: 5'-CGCACCACTGATACCCT-3' | 1044-1150 |  |  |  |
| *Cdc2* | F: 5' –GGGCACTCCCAATAA-3' | XM_572099 | 60 | 93 | 34 |
|  | R: 5'-GATGCTAGGCTTCCTG-3' | 631-723 |  |  |  |
| *Bcl-2* | F: 5'-GCCTTCTTTGAGTTCGGTGGG-3' | DQ926871 | 60 | 115 | 34 |
|  | R: 5'-TGTGCAGGTGCCGGTTCAG-3' | 938-1052 |  |  |  |
| *Bax* | F: 5'-GATTGCCGCCGTGGAC-3' | DQ926869 | 60 | 88 | 34 |
|  | R: 5'-GCCCCAGTTGAAGTTGC-3' | 306-393 |  |  |  |
| *Bcl-xL* | F: 5'-GGCAACCCATCCTGGCACCT-3' | AY263145 | 60 | 159 | 34 |
|  | R:5'-AACTCGTCGCCTGCCTCCCT-3' | 118-276 |  |  |  |
| *P21* | F: 5'-ACTGTCTTGTACCCTTGTGCC-3' | XM_003950827 | 60 | 108 | 34 |
|  | R: 5'-AAATCTGTCATGCTGGTCTGC-3' | 572-679 |  |  |  |
| *P27* | F: 5'-GGCTCCGGCTAACTCTGA-3' | XM_522347 | 60 | 157 | 34 |
|  | R: 5'-TTCTTCTGTTCTGTTGGCTCTT-3' | 1081-1237 |  |  |  |
| *β-catenin* | F: 5'-GCTTGGAATGAGACTGCTGA-3' | X87838 | 60 | 114 | 34 |
|  | R: 5'-CTGGCCATATCCACCAGAGT-3' | 2221-2334 |  |  |  |
| *cyclinD1* | F: 5'-TGCCACAGATGTGAAGTTCATT-3' | NG_000002 | 60 | 162 | 34 |
|  | R: 5'-CAGTCCGGGTCACACTTGAT-3' | 776-937 |  |  |  |
| *Survivin* | F: 5'-TTCTCAAGGACCACCGCATC-3' | DQ508252 | 60 | 162 | 34 |
|  | R: 5'-AGCCTTCCAGCTCCTTGAAG-3' | 159-320 |  |  |  |
| *ING5* | F: 5’- GGGAGATGATTGGCTGTG-3’ | NM_032329.4 | 60 | 146 | 34 |
|  | R: 5’-CCTTTGGGTTTCGTGGTA-3’ | 614-759 |  |  |  |
| *mToR* | F: 5'-CGCTGTCATCCCTTTATC-3' | NM_004958 | 60 | 96 | 34 |
|  | R: 5'-TTCTTCTCCCTGTAGTCCC-3' | 2092-2187 |  |  |  |
| *C-myc* | F: 5'-AGCGACTCTGAGGAGGAACA-3' | X00676 | 60 | 108 | 34 |
|  | R: 5'-TCCAGCAGAAGGTGATCCA-3' | 1318-1425 |  |  |  |
| *14-3-3* | F: 5'-CAAAGACAGCACCCTCA-3' | XM_010379682.1 | 60 | 91 | 34 |
|  | R: 5'-TTCTGCCGCATCACAT-3' | 845-935 |  |  |  |
| *BTG3* | F: 5’-GCAGTTGAGAGGT TTGCTGA-3’ | NM_ 01130914.1 | 60 | 451/394 | 34 |
|  | R: 5’-TAACTTTCCTGGAGATCTCATT- 3’ | NM_006806.4 |  |  |  |
| *Beclin1* | F: 5'-GATGGAAGGGTCTAAGACGTCCAA-3' | XM_010385905.1 | 60 | 160 | 34 |
|  | R: 5'-TTTCGCCTGGGCTGTGGTAAG-3' | 145-304 |  |  |  |
| *Akt1* | F: 5'-TCTTTGCCGGTATCGTGT-3' | F283818S13 | 60 | 150 | 34 |
|  | R: 5'-TGTCATCTTGGTCAGGTGGT-3' | 1559-1708 |  |  |  |
| *Raptor* | F: 5' –GGAGCGCCCGCTATTT-3' | BC136654 | 60 | 161 | 34 |
|  | R: 5'-GTCGTCCAATCTCGTAATGC-3' | 3690-3850 |  |  |  |
| *Bad* | F: 5' –GAGGACGACGAAGGGATGG-3' | XM_004051439 | 62 | 85 | 34 |
|  | R: 5'-GCTGTGCTGCCCAGAGGTT-3' | 119-203 |  |  |  |
| *CyclinE2* | F: 5'-ATTTGGCTATGCTGG-3' | XM_017013959.1 | 48 | 101 | 34 |
|  | R: 5'-GCTCTTCGGTGGTGT-3' | 1436-1536 |  |  |  |
| *Atg1* | F: 5'-CCTGCTGAGCCGAGAATGG-3' | XR_001748883.1 | 60 | 227 | 34 |
|  | R: 5'-CCTGCTTCACAGTGGACGACA-3' | 124-350 |  |  |  |
| *DKK3* | F: 5'-CACCCTCAATGAGATGTTCC-3' | NM_001330220.1 | 60 | 161 | 34 |
|  | R: 5'-TGGTCTCATTGTGATAGCTG-3' | 260-420 |  |  |  |
|  |  |  |  |  |  |
